# Early-onset breast cancer patients in the South and Southeast of Brazil should be tested for the *TP53* p.R337H mutation

**DOI:** 10.1590/1678-4685-GMB-2014-0343

**Published:** 2016-05-24

**Authors:** Kelvin C. Andrade, Karina M. Santiago, Fernanda P. Fortes, Lisley I. Mambelli, Amanda F. Nóbrega, Maria I. Achatz

**Affiliations:** 1 Centro Internacional de Pesquisa e Ensino, A.C. Camargo Cancer Center, São Paulo, SP, Brasil; 2 Departamento de Oncogenética, A.C. Camargo Cancer Center, Sao Paulo, SP, Brazil

**Keywords:** Breast cancer, Li-Fraumeni syndrome, p.R337H, TP53

## Abstract

Germline *TP53* mutations are associated with Li-Fraumeni syndrome (LFS), a disease that predisposes carriers to a wide variety of early onset tumors. In southern and southeastern Brazil, a high frequency of a germline *TP53* mutation, p.R337H, was diagnosed in 0,3% of the population due to a founder effect. Carriers are at risk for developing cancer but the penetrance is lower than in typical DNA binding domain mutations. To date, only a few families were detected and diagnosis of carriers remains a challenge. Therefore, the inclusion of additional criteria to detect p.R337H carriers is necessary for the Brazilian population. We assessed the A.C. Camargo Cancer Center Oncogenetics Department database in search of common characteristics associated with p.R337H families that did not fulfill LFS/LFL clinical criteria. Among 42 p.R337H families, three did not meet any LFS/LFL criteria. All cases were young female patients with breast cancer diagnosed before age 45 and with no family history of LFS linked-cancers. Our results suggest that screening for the germline *TP53* p.R337H mutation should be indicated, along with *BRCA1* and *BRCA2* genetic testing, for this group of patients, especially in the South and Southeast of Brazil.

Li-Fraumeni syndrome (LFS, OMIM #151623) is a rare autosomal dominant genetic disorder inherited by germline *TP53* mutations (Malkin *et al.*, 1990). Carriers are predisposed to the development of a wide variety of early onset tumors, especially to those denominated as LFS core tumors: premenopausal breast cancer, soft-tissue sarcoma (STS), central nervous system tumors (CNS), and adrenocortical carcinomas (ADR) (Li and Fraumeni, 1969a, b).

In order to identify at-risk families who carry these mutations, different criteria for clinical diagnosis have been established (Table 1). Since its publication, classical criteria have been modified due to the presence of families which, although not fulfilling the definition, were positive for germline *TP53* mutations. This group of patients belongs to a variant form of LFS, named Li-Fraumeni-*like* (LFL), which is defined by either more inclusive parameters or additional criteria (Birch *et al.*, 1994; Chompret *et al.*, 2001; Eeles, 1995; Tinat *et al.*, 2009).

**Table 1:** Clinical criteria for LFS diagnosis

Criteria	Description	Reference
*Classic*	• Proband diagnosed with a STS before age 45 AND; - One first-degree relative with any tumor diagnosed before age 45 AND; - Another first- or second-degree relative diagnosed with any cancer before age 45 or a STS at any age	Li and Fraumeni, 1988
*Birch*	• Proband with a cancer diagnosed in childhood (STS, CNS, ADR) OR; • Proband diagnosed with a STS, SNC, or ADR before age 45 AND; - One first- or second-degree relative diagnosed with a tumor from the LFS spectrum at any age AND; - First- or second-degree relative diagnosed with any cancer before age 60	Birch, 1994
*Eeles*	*• LFL-Eeles 1* - Two first-or second- degree relatives diagnosed with a typical LFS tumor at any age *• LFL-Eeles 2* - Proband diagnosed with a STS at any age AND; - Two first-or second-degree relatives diagnosed with two different typical LFS tumors at any age	Eeles, 1995
*Chompret*	• Proband diagnosed with a typical LFS tumor before age 36 AND; - One first- or second-degree relative diagnosed with any cancer before age 46 OR; - One relative with multiple tumors diagnosed at any age • Proband with multiple primary tumors - including two typical LFS tumors - with the first diagnosed before age 36 regardless of family history • Proband with ADR at any age regardless of family history	Chompret, 2001
*Chompret 2009*	• Proband with a typical LFS tumor diagnosed before age 46 AND; - At least one first- or second-degree relative diagnosed with a typical LFS tumor (except breast cancer if the proband is/was affected by breast cancer) before age 56 or with multiple primary tumors OR; • Proband with multiple primary tumors (other than multiple breast tumors) - including at least two from LFS tumor spectrum - with the first diagnosed before age 46; • Proband diagnosed with ADR or CPC at any age irrespective of family history	Tinat, 2009

Abbreviations: STS: soft-tissue sarcoma; CNS: central-nervous system tumors; ADR: adrenocortical carcinoma; CPC: choroid-plexus carcinoma

Interestingly, a specific germline *TP53* mutation (NC_000017.9: c.1010G > A; p.R337H) was reported as highly associated with LFS/LFL families in Brazil (Achatz *et al.*, 2007). It is present in 0,3% of the local population from southern and southeastern regions of the country (Palmero *et al.*, 2008; Custódio *et al.*, 2013) due to a founder effect (Pinto *et al.*, 2004; Garritano *et al.*, 2010). One of the hypotheses to explain why this deleterious mutation has persisted is based on its relatively reduced penetrance, which confers a tumor risk of 30% before the age of 30, while lifetime cancer risk is similar to other *TP53* mutations (Garritano *et al.*, 2010). Thus, most carriers may have their children before developing cancer, spreading the mutation throughout generations. Also, the tumor profile among Brazilian carriers is similar to that of DNA-binding domain mutations found elsewhere in the world, but with some age difference and a higher risk for other types of tumors. In spite of its elevated prevalence, appropriate criteria to identify carriers, as well as guidelines to facilitate and direct genetic testing are still missing and, therefore, the number of carriers may be underestimated. Hence, our aim was to investigate the family history of p.R337H carriers who did not fulfill any of the LFS/LFL criteria, and define when individuals without criteria would benefit from testing for p.R337H.

This study is based on the A.C. Camargo Cancer Center Oncogenetics Department's database. The department has been following patients at high-risk for cancer development since 1999 and currently comprises 7,059 individuals from 607 families. For each family we obtained a detailed family history regarding tumor diagnosis and clinical data for both index patients and their relatives. Patients eligible for either *TP53* sequencing or point-mutation directed genetic testing are also registered in this database. From 348 families tested for germline *TP53* mutations, 42 were found to carry the p.R337H mutation.

Table 2 shows the number of families that fulfilled each of the LFS/LFL criteria. From the 42 families identified as p.R337H carriers, three did not meet any of the LFS/LFL criteria. According to their respective pedigrees (Figure 1), family Y0347 (Figure 1A) presented only two cases of maligancy: the proband with an invasive ductal carcinoma (IDC) diagnosed at the age 41 and her paternal uncle with prostate cancer at the age 60, which is not considered as an LFS-core tumor. Family Y0348 (Figure 1B) also presented cases of early-onset breast cancer; an IDC and a ductal carcinoma *in situ* (DCIS) diagnosed in the proband at the ages of 42 and 46, respectively, in addition to a breast cancer diagnosed in her mother at the age 61. Finally, the pedigree of family Y0349 (Figure 1C) includes a proband diagnosed with breast cancer at the age of 29 and cases of uterus and prostate cancers in her second- and third- degree relatives.

**Table 2:** Families carriers of the p.R337H mutation distributed according to different LFS criteria.

LFS/LFL Criteria	Number of families
Classic	1
Birch	13
Eeles	
Eeles 1	10
Eeles 2	5
Chompret	
Chompret 2001	7
Chompret 2009	3
None	3
Total	42

**Figure 1:**
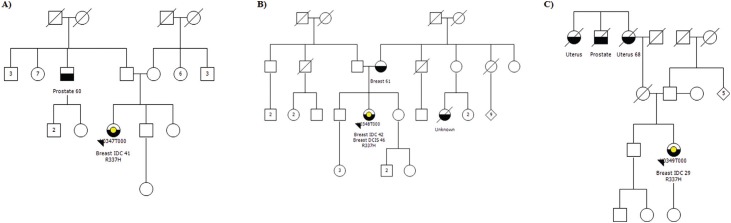
Pedigrees of the families affected by the germline *TP53* p.R337H mutation. A) Family Y0347, B) Family Y0348, C) Family Y0349. The proband is indicated by an arrow; black symbol: patient affected by malignant tumor; yellow symbol: patient carrier of the p.R337H mutation; first information below the symbol: type of tumor and age at diagnosis.

Altogether, we identified three different p.R337H families that did not fulfill any of the clinical criteria for LFS diagnosis. The main common observations in these families were the cases of breast cancer, diagnosed before age 45, irrespective of family history.

It has been suggested that women diagnosed with breast cancer before age 30, along with a family history of one or more core LFS cancers in a first- or second-degree relative should also be considered for *TP53* genetic testing. Under this premise, Gonzalez *et al.* (2009) found a likelihood of 100% (5 of 5) individuals harboring a germline *TP53* mutation. In contrast, the authors did not detect any mutation carrier in the group composed by 15 women diagnosed with invasive ductal carcinoma between the interval of 30-49 years and who did not have any core LFS tumor in the family history. Similar results were described later (Mouchawar *et al.*, 2010), and the probability of identifying a germline *TP53* mutation in women diagnosed with early onset breast cancer and who have a negative family history was defined as ranging from 5% to 8%, (McCuaig *et al.*, 2012).

The three families detected in our study presented some features that should be carefully interpreted based on specific p.R337H characteristics. Different from the findings described by Gonzalez *et al.* (2009), two positive cases (Y0347T000 and Y0349T000) did not have any core LFS tumor in their first- or second-degree relatives. In addition, although the family Y0348 includes two cases of breast cancer, it did not meet any of the LFS criteria due to the relatively older age at tumor diagnosis of the proband's mother. These particularities could be consequences of the low penetrance presented by the p.R337H mutation, especially before the age of 30 (Garritano *et al.*, 2010), which raises the possibility of later-than-expected ages at cancer development when compared to those described in currently applied LFS clinical criteria. Therefore, this might be a plausible explanation for both the absence of other affected individuals in the pedigree, as well as a slightly older age at cancer onset.

The indication of simultaneous genetic testing for *BRCA1/BRCA 2* and *TP53* has been proposed especially for women with breast cancer diagnosed before age 35 who have a family history of LFS-linked cancers (Lee *et al.*, 2012). Conversely, Tinat *et al.* (2009) suggested *TP53* testing only for women diagnosed with early onset breast cancer who are negative for mutations in *BRCA1* and *BRCA2*, irrespective of family history. Nonetheless, the authors state that it should be avoided in those who do not present a family history of cancer or multiple primary tumors, mainly due to the low estimated prevalence of positive cases in this category (less than 5%) and the psychosocial burden induced by a *TP53* genetic testing. In accordance with our observations, Gomes *et al.* (2012) described two p.R337H carriers diagnosed with breast cancer before the age 40 in an unselected breast cancer-cohort with 390 participants (0,5%), indicating that the genetic testing for the p.R337H mutation could potentially be included in existing screening panels. Similarly, Giacomazzi *et al.* (2014) investigated the prevalence of the p.R337H mutation in two different Brazilian groups of women diagnosed with breast cancer: one composed by affected individuals with a family history compatible with hereditary breast cancer but no LFS/LFL features and another one, by women unselected for family history The authors found mutation frequencies of 3,4% and 8,6% for each group, respectively. Due to this frequency, they proposed that this mutation may play an important role in the incidence of breast cancer in Brazil.

These findings, along with ours, strengthen the importance of suggesting concomitant *TP53* p.R337H genetic testing for women affected by breast cancer before age 45, irrespective of family history, particularly in the South and Southeast of Brazil, where the prevalence of a germline *TP53* is considerably higher than elsewhere in the world. The inclusion of this group of patients would potentially avoid LFS/LFL underdiagnosis and inappropriate genetic counseling.
